# Larger lakes are hit harder and longer by *Dreissena* invasion

**DOI:** 10.1007/s10530-026-03964-7

**Published:** 2026-10-23

**Authors:** Alexander Y. Karatayev, Vadim A. Karatayev, Lyubov E. Burlakova, Lars G. Rudstam, James M. Watkins, Katya E. Kovalenko, Alexander L. Koeberle, Salomé E. Boudet, Bas W. Ibelings, Nikolai V. Barulin, Nicole Gallina, Jonas Mauch, Alexandra A.-T. Weber, Holly Waterfield, Matthew Albright, Piet Spaak

**Affiliations:** 1 Great Lakes Center, SUNY Buffalo State University, Buffalo, NY 14222 USA; 2 Department of Aquatic Ecology, Swiss Federal Institute of Aquatic Science and Technology (Eawag), 8600 Dübendorf, Switzerland; 3 Department of Biology, University of Maryland, College Park, MD USA; 4 Cornell Biological Field Station at Shackelton Point, Ashley School of Global Development and the Environment, Cornell University, Bridgeport, NY 13030 USA; 5 Natural Resources Research Institute, University of Minnesota, Duluth, MN 55811 USA; 6 Department FA Forel for Environmental and Aquatic Sciences, Institute for Environmental Sciences, University of Geneva, Geneva, Switzerland; 7 International Commission for the Protection of the Waters of Lake Geneva (CIPEL), Geneva, Switzerland; 8 Department of Community and Ecosystem Ecology, Leibniz Institute of Freshwater Ecology and Inland Fisheries (IGB), 12587 Berlin, Germany; 9 Biology Department, Oneonta Biological Field Station, SUNY at Oneonta, Oneonta, NY USA

**Keywords:** Invasive species, Quagga mussel, Zebra mussel, Long-term data, Lakes, Ecological impact

## Abstract

Invasive quagga mussels are swiftly spreading across Europe and North America, causing major ecological and economic impacts even in lakes already invaded by zebra mussels. A key challenge is to understand the magnitude and timing of mussel impacts across a wide range of freshwater ecosystems, to predict potential impacts on uninvaded systems. We assembled high-resolution data sets spanning 18 North American and European invaded lakes and five key ecosystem variables (Secchi depth, phosphorus and chlorophyll concentration, phytoplankton and zooplankton biomass). We then address the questions: How have the pace and magnitude of dreissenid impacts varied with lake morphometry (area, depth, volume)? How have impacts differed between polymictic and stratified lakes? Which lakes have exhibited recovery following zebra and quagga mussel invasions? We found that impacts manifest earlier in polymictic than in stratified lakes. After the initial impacts, lakes invaded by zebra mussels usually exhibited signs of partial ecosystem recovery, which was interrupted in lakes that experienced a secondary invasion by quagga mussels. In the Laurentian Great Lakes, mussel populations reached maximum biomass later, and ecosystem impacts manifested more slowly but culminated at a higher overall impact level. Furthermore, no ecosystem recovery in the Great Lakes has been observed to date, even 30 years after mussel invasions, though recovery may be delayed in large lakes. Our results indicated that lake size is more important than other parameters in determining *Dreissena* impact, and findings from smaller inland lakes cannot be directly applied to larger systems (e.g., the Great Lakes) and vice versa.

**Supplementary Information:**

The online version contains supplementary material available at https://doi.org/10.1007/s10530-026-03964-7.

## Introduction

Non-native species can cause a wide range of impacts from none to severe declines in biodiversity and disruption of ecosystem functions (Gurevitch and Padilla 2004; Strayer 2010; Lopes-Lima et al. 2025). Invasive bivalves *Dreissena polymorpha* (Pallas 1771), the zebra mussel, and *D. rostriformis bugensis* (Andrusov 1897), the quagga mussel, are considered the most aggressive freshwater invaders in the Northern hemisphere (reviewed in Higgins and Vander Zanden 2010; Karatayev and Burlakova 2025a, b). Both species spread rapidly across landscapes, become extremely abundant in invaded waterbodies, and are highly efficient suspension feeders—features typical of marine mussels but representing a novel ecological type in recently invaded freshwaters (Karatayev et al. 2015; Pollux et al. 2010; Karatayev and Burlakova 2025a). As a result, invasive dreissenids often have profound ecosystem impacts, including increases in water transparency and declines in chlorophyll and seston concentrations in the water column, along with changes in nutrient concentrations and cycles, as well as substantial changes in aquatic community biomass and structure (Higgins and Vander Zanden 2010; Li et al. 2021; Karatayev and Burlakova 2025a).

The magnitude of *Dreissena* spp. effects in individual lakes depends on many biotic and abiotic factors, including mussel density and distribution, water mixing regimes, retention time, extent of anoxia, lake morphology, and time since the initial invasion (Karatayev et al. 2002; Reed-Andersen et al. 2000; Karatayev and Burlakova 2025a). During the initial phase of invasion, rapid *Dreissena* spp. population growth causes profound changes in the invaded ecosystems (Vanderploeg et al. 2002; Higgins and Vander Zanden 2010; Karatayev and Burlakova 2025a). Later in the invasion, the ecosystem may stabilize or recover due to the invader population decline (Simberloff and Gibbons 2004; Karatayev et al. 2015; Strayer et al. 2017), resilience in community structure, density-dependent changes in the abundance of the invader, or evolutionary adaptations (learning, eco-evo dynamics) by resident species to the new invader (Karatayev et al. 2023, 2025). However, quagga mussels are swiftly spreading across Europe and North America, colonizing lakes already invaded by zebra mussels and leading to additional ecological and economic impacts (Pollux et al. 2010; bij de Vaate et al. 2014; Haltiner et al. 2022; Karatayev and Burlakova 2025a).

In a previous study, we used 35-year datasets from seven shallow, polymictic lakes to investigate longer-term changes in these ecosystems following the invasion of dreissenids (Karatayev et al. 2023). The analysis revealed remarkably similar trends across these lakes, with the strongest ecosystem impacts occurring within 5–10 years of zebra mussel invasion. Plankton communities then exhibited a partial recovery, which we define henceforth as a return towards pre-invasion ecosystem conditions. However, this recovery was reversed or absent in lakes subsequently invaded by quagga mussels (Karatayev et al. 2023). Here we ask if these impacts and responses to dreissenids also occur in monomictic and dimictic (stratified) lakes and in very large lake ecosystems such as the Laurentian Great Lakes.

In stratified lakes, dreissenid impacts could be especially strong because quagga mussels have the potential to move deeper, colonize the entire bottom down to the deepest areas, and reach extremely high densities (Karatayev et al. 2021; Karatayev and Burlakova 2025a). However, the bulk of the quagga mussel population is located below the thermocline in stratified lakes and is isolated from surface waters for most of the growing season. Lower temperatures below the thermocline will also decrease mussel feeding rates (Karatayev et al. 2018a; Haltiner et al. 2023). Therefore, their impact on planktonic communities may be lower than in polymictic lakes (Karatayev and Burlakova 2025a). Further, the time between the initial invasion and when the ecosystem stabilizes and begins to recover may depend on lake size and could be significantly longer in the Laurentian Great Lakes (Great Lakes hereafter) than in smaller inland lakes (Karatayev and Burlakova 2025a, b), thereby delaying both the manifestation of mussel impacts and any ecosystem recovery toward pre-invasion conditions. In addition, factors such as land-use changes, changes in agricultural practices, and warming temperatures may accelerate or slow dreissenid impacts on invaded ecosystems and may confound mussel impacts.

Here, we assemble high-resolution data sets spanning five key ecosystem features across 18 lakes in the USA, Canada, Belarus, Hungary, the Netherlands, Denmark, Switzerland, and Germany, all invaded by dreissenid mussels (Online Resource 1). We analyze changes shared among lakes over time since the invasion to robustly attribute specific ecosystem indicator changes to define the impacts of dreissenid introduction, tracking time since invasion and lake morphometry. We begin with the question: does lake morphometry affect *Dreissena* population growth and distribution? Then we compare *Dreissena* impacts in inland polymictic and stratified (mono- or dimictic) lakes with the Great Lakes to determine whether lake size is more important than lake depth in the timing and magnitude of *Dreissena* impacts. Finally, we investigate whether ecosystem recovery following *Dreissena* invasions occurs in all lakes, whether trajectories follow similar paths among lake types, and whether lakes respond differently to zebra versus quagga mussel invasions.

## Materials and methods

We study long-term trends using data from 18 invaded lakes, including nine inland polymictic lakes and six stratified inland lakes in North America and Europe, and three stratified North American Great Lakes (Table 1; Online Resource 1). For clarity, we refer to lakes smaller than the Great Lakes as “inland lakes” throughout the text. Following Karatayev et al. (2023), we analyze standardized changes in phytoplankton (chlorophyll and biomass), zooplankton, and abiotic variables (Secchi depth and total phosphorus) as a function of time since the dreissenid invasion. These trends provide evidence of both impacts and recovery. We exclude macrophyte and zoobenthic data from our analysis because most stratified lakes lack this information. Importantly, we define the year of initial invasion as the first year in which detectable dreissenid biomass was recorded in the lake (thereafter, the initial invasion). This date may lag 1–3 years behind the actual introduction of the first mussels in some lakes, while in others, the date of first introduction is unknown. We therefore use the year of first record as the most consistent and tangible proxy for invasion timing across lakes, while acknowledging that this may not reflect the exact same invasion stage in every lake. All lakes have long-term dreissenid presence and span a wide range of mean mussel wet biomass (Table 2, though not all lakes have actual biomass data), lake depth (1.2–154 m mean depth), surface area (7.4–53,537 km^2^), and water retention time (0.14–95 years) (Table 1).

**Table 1:** Physical characteristics of the studied lakes and data sources for ecosystem impacts used in the analysis

Lake, country	Surface, km^2^	Maximum depth, m	Average depth, m	Retention time, year	Data source
*Polymictic inland lakes*					
Lukomskoe, Belarus	36.7	11.5	6.7	7.0	Karatayev et al. (2023)
Naroch, Belarus	79.6	24.8	9.0	8.0	Karatayev et al. (2023)
Myastro, Belarus	13.1	11.3	5.4	2.5	Karatayev et al. (2023)
Oneida, USA	207.0	16.8	6.8	0.64	Karatayev et al. (2023)
Balaton (eastern basin), Hungary	218	12.2	3.7	NA	Karatayev et al. (2023)
Veluwe, The Netherlands	31.3	5.0	1.8	NA	Noordhuis et al. (2016)
Eem, The Netherlands	15.2	4.0	2.1	NA	Noordhuis et al. (2016)
Hinge, Denmark	9.3	1.3	1.2	3.1	Søndergaard et al. (2025)
Müggelsee, Germany	7.4	8.0	4.9	0.14	Wegner et al. (2019); Mauch et al. (2026); IGB (2026)
*Stratified inland lakes*					
Simcoe, Canada	725	41	15	16	Lake Simcoe Monitoring (2025)
Seneca, USA	175	188	89	18	Halfman (2012)
Otsego, USA	16.4	51	25	3	Waterfield and Albright (2023)
Keuka, USA	47.5	56	31	7	Keuka Lake Association; Callinan (2001); Koeberle (2025)
Constance, Switzerland/Germany/Austria	473	251	101	4.5	IGKB (2025)
Geneva, Switzerland/France	580	310	154	12	CIPEL (2025); Rimet et al. (2020)
*Stratified Great Lakes*					
Ontario, USA/Canada	18,960	244	86	6.2	GLENDA (2025)
Michigan, USA	53,537	282	90.5	95.2	GLENDA (2025)
Huron, USA/Canada	43,086	229	66	15.2	GLENDA (2025)

**Table 2:** Year of the first record, lag time between first record and population maximum, and average wet biomass (g/m^2^) of dreissenids in the studied lakes

Lake	First record	Maximum, years	Biomass	First record	Maximum, years	Biomass	Data source
	*Dreissena polymorpha*			*D. rostriformis bugensis*			
*Polymictic inland lakes*							
Lukomskoe	1972	3	262 (1972–1989)	None			Karatayev et al. (2023)
Naroch	1989	4	114 (1993–2016)	None			Karatayev et al. (2023)
Myastro	1984	NA	409 (1993–2017)	None			Karatayev et al. (2023)
Oneida	1991	2	804 (1992–2006)	2005	7	1178 (2007–2023)	Hetherington et al. (2019), Karatayev et al. (2025)
Balaton (eastern basin)	1932	2	293 (2001–2005)	2008	5	1310 (2008–2017)	Karatayev et al. (2011, 2023)
Veluwe	1995	NA	NA	2009	5	NA	Noordhuis et al. 2016)
Eem	1995	NA	149 (2004–2013)	2009	7	364 (2013)	Noordhuis et al. (2016)
Hinge	2006	NA	NA	None			Søndergaard et al. (2025)
Müggelsee	19th cent	NA	113 (2011)	2011	7	1130 (2017)	Arndt et al. (1993), Wegner et al. (2019)
*Stratified inland lakes*							
Simcoe	1992	3	196 (2009, 215)	2004	6	424 (2009, 2015)	Evans et al. (2011), Ginn et al. (2018)
Seneca	1992	NA	0 (2025)	1994	6	519 (2025)	Watkins et al. (2012), Our data
Otsego	2007	NA	NA	2017	6	228 (2021–2024)	Beck et al. (2023); Harman (2023), Our data
Keuka	1994	NA	173 (2010)	2008	NA	412 (2010, 2025)	Watkins et al. (2012); Our data
Constance	1960s	NA	NA	2016	6	320 (2021–2025)	SeeWandel (2025), IGKB (2025)
Geneva	1960s	NA	NA	2015	7	842 (2022, 2024)	Eawag; Unige; CIPEL (2025); Salomé Boudet
*Stratified Great Lakes*							
Ontario	1989	5	40 (1990–1995)	1990	28	377 (1995–2018)	Karatayev et al. (2021, 2022)
Michigan	1989	4	12 (1994–2000)	1997	24	648 (2005–2025)	Karatayev and Burlakova (2025c)
Huron	1989	NA	15 (2000)	1997	20	184 (2003–2022)	Karatayev and Burlakova (2025c)

In parentheses are the years of dreissenid biomass observation. NA—no data available

For all lakes, whenever possible, we collected available information from peer-reviewed and grey literature (e.g. technical reports, abstracts, proceedings, etc.), electronic data sources, unpublished reports and archive materials as well as authors' unpublished data on water transparency (Secchi depth, m), total phosphorus (TP) concentration (μg/L), chlorophyll *a* concentration (μg/L), wet phytoplankton biomass (g/m^3^), wet zooplankton biomass (g/m^3^), except lakes Simcoe, Müggelsee, Constance, and Geneva where zooplankton densities were used (crustaceans in Lake Simcoe and crustaceans and rotifers in the other three lakes), and *Dreissena* spp. wet biomass (tissue and shells, g/m^2^). Whenever possible, we used monthly data averaged over the growing season (April–October), although the Great Lakes were sampled only twice a year (in April and August). All data used in the analysis are provided in the supplement along with the sampling design and data sources for each lake (Online Resource 1). All lakes studied were initially colonized with zebra mussels, and later 14 of them were invaded with quagga mussels, including four lakes where zebra mussel invasion occurred 75–100 years before quagga invasion (Table 2) and long before monitoring began, making it harder to tease apart the impacts of the two species.

### Modeling framework

Throughout our analysis, we resolved the impact of each *Dreissena* species introduction on each ecosystem variable *x* using a Generalized Additive Model (GAM) framework (Online resource 2. Code to run analyses). We first standardized observations in each lake *i* and year *t* into z-scores as ${x}_{i,t}^{z}=({x}_{i,t}- \overline{{x}_{i}})/{s}_{x,i}$. To account for both direct effects of invader biomass/activity and delayed ecosystem responses, we then modeled invasion effects on standardized variable time series *x*^*z*^ across all lakes as a smooth function of time since *Dreissena* invasion using the package mgcv (Wood 2017) in program R version 3.5.2. Throughout, time since invasion refers to the years since (a) the detection of zebra mussels or (b) the detection of quagga mussels in lakes where zebra mussels were already present but sparse. This approach allows us to zero in on the ecosystem effects of a large increase in dreissenid biomass.

To model how invasion impacts depend on lake size, as a visualization, we first fit two-dimensional splines of ecosystem impacts as a function of (1) years since invasion in the monitoring survey and (2) log lake area. We assumed and verified in preliminary analyses a normal error distribution. Based on the Akaike Information Criterion (AIC) score, log lake surface area outperformed two alternative metrics—log volume and log mean depth—as a predictor of mussel impacts on every ecosystem variable. Therefore, we use "lake size" to refer to the log of lake area henceforth; throughout, "log" refers to the natural logarithm. The area of our study systems clustered roughly into three groups of lakes: polymictic lakes (the smallest), stratified lakes (intermediate size), and the Great Lakes.

Next, we quantified differences in dreissenid invasion impacts over time among lake types by fitting one-dimensional splines of year since invasion, which were hierarchical by lake type, using restricted maximum likelihood (Pedersen et al. 2019). Specifically, we used thin plate regression splines (‘TPRS’) with a penalty on the squared first derivatives (i.e., marginal TPRS basis for lake type penalizes the squared first derivatives). Given our focus on resolving general invasion impacts, we minimized lake-specific effects for each ecosystem variable *x*^*z*^ by selecting the maximum basis dimension *k*^*x,z*^ of each hierarchical spline using the Bayesian information criterion (BIC). Specifically, we initialized each spline with *k*=3 (corresponding to a linear model) and progressively increased *k* by 1 until the next *k* increase decreased BIC by less than two; all resulting values of *k*^*x,z*^ were 6 or less. To test whether lake size significantly affected invasion impacts on each ecosystem feature, we compared the performance of a model with the impact modeled hierarchically by lake group (polymictic, stratified, or Great Lake) *versus* a non-hierarchical model that assumed identical invasion impacts in all lake groups using the AIC. Hierarchical models that improved performance by >2 ΔAIC indicate that impacts vary significantly by lake group.

To compare *Dreissena* impacts across lake types, we calculated the mean change predicted by hierarchical splines over years 17–21 since invasion, along with its 97.5% confidence interval, for each variable and lake type. This time range was selected because 72% of lakes had >16 years of post-invasion data, but this proportion quickly declines beyond 20 years since invasion. As a conservative check for differences, we noted a difference between two lake types only when their 97.5% confidence intervals excluded each other’s mean. We used 97.5 rather than 95% confidence intervals to account for pairwise tests among the three lake groups.

In plotting invasion impacts by lake group, we re-centered the fitted group-specific splines such that the impact was zero at time zero for each ecosystem variable. In analyzing the fitted splines, we approximated the timing and direction of *Dreissena* effects based on the slopes of fitted splines calculated using the package 'tsgam,' where negative (positive) slopes indicate a decrease (increase) in the ecosystem variable. We conservatively determined decreases (increases) in an ecosystem variable at year *t* as significant when the 95% confidence intervals of the slope at year *t* were (1) below (above) zero and (2) remained so for at least four consecutive years. For year 'zero' (before the *Dreissena* invasion), if available, we averaged data across 5 years immediately preceding the invasion.

## Results

### Lake morphometry governs Dreissena population growth and distribution

**Fig. 1:**
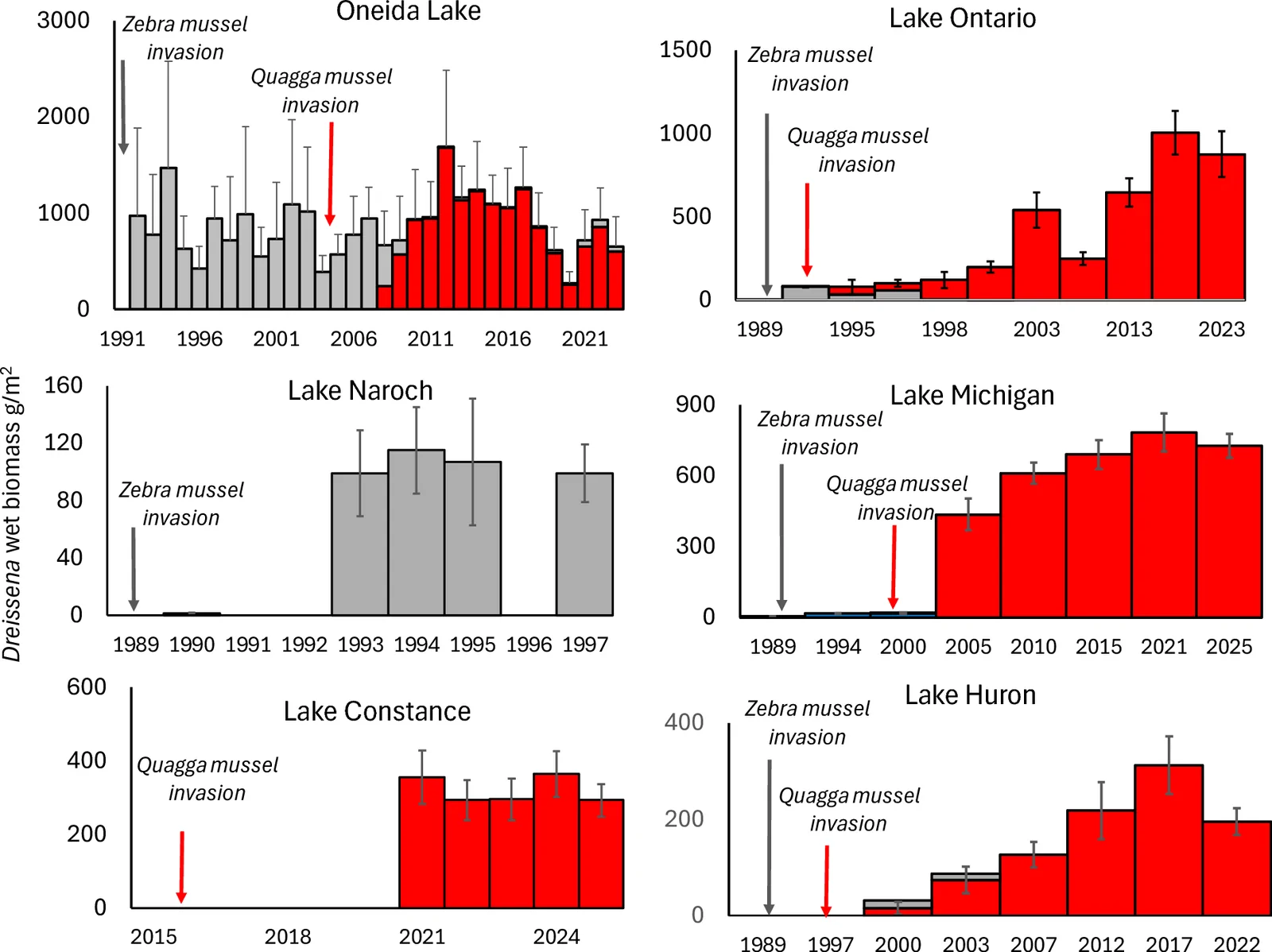
Population dynamics of *Dreissena polymorpha* (zebra mussel, grey bars) and *D. rostriformis bugensis* (quagga mussel, red bars) wet biomass (g/m^2^) in inland polymictic lakes (Oneida and Naroch), inland stratified lakes (Constance), and in the Great Lakes (Ontario, Michigan, and Huron). Vertical lines mark one standard error. For the Great Lakes, standard errors were estimated for the quagga mussel. The year of the first record of the zebra and quagga mussel in each lake is indicated

We found that lake size (area) was the most important factor determining dreissenid population dynamics and species distribution within the waterbody. In inland polymictic and stratified lakes, zebra mussel populations reach their maximum significantly earlier than quagga mussel populations (2.8 ± 0.4 vs. 6.1 ± 0.3 years) (Table 2). For example, the time from first detection to population maximum for zebra mussels was 4 years in Lake Naroch and 2 years in Oneida Lake, while the time from first detection to population maximum for quagga mussels was 7 years in Oneida Lake (Fig. 1) and 6 years in Lake Constance. We note that quagga mussels continue to grow in the deep offshore zone of Lake Constance, but this does not contribute significantly to the total biomass.

**Fig. 2:**
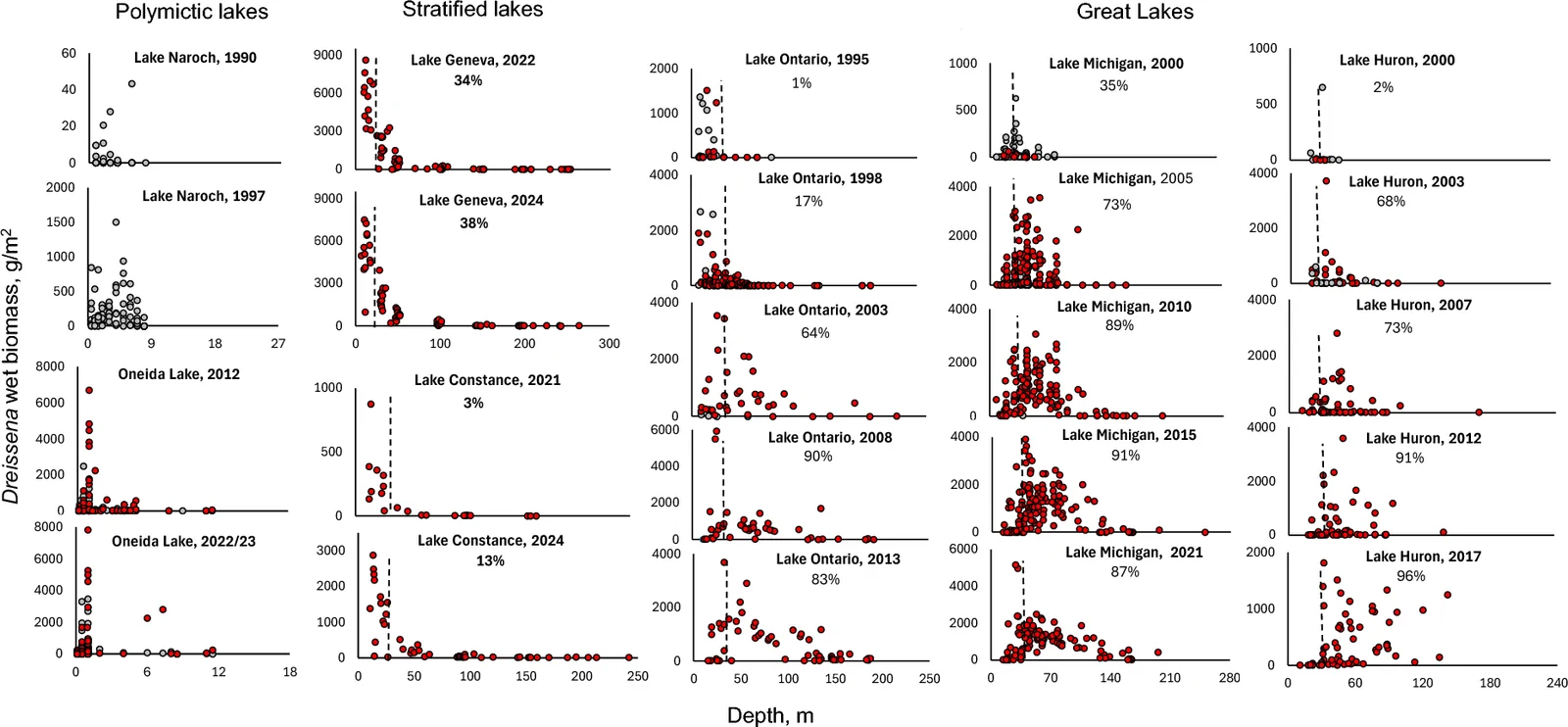
Depth distribution of *Dreissena polymorpha* (grey circles) and *D. rostriformis bugensis* (red circles) wet biomass (g/m^2^) in polymictic lakes (Naroch and Oneida), inland stratified lakes (Constance and Geneva), and Great Lakes (Ontario, Michigan, and Huron). The dashed line indicates the maximum depth of the epilimnion: 20 m in lakes Constance and Geneva, and 30 m in the Great Lakes. The number with % indicates the percentage of mussel biomass located below the thermocline

In contrast, it took quagga mussels at least three times longer to reach a lake-wide biomass maximum in the Laurentian Great Lakes than in inland lakes (5–7 in inland lakes *versus* 20–28 years after first being recorded in the Great Lakes) (Table 2; Fig. 1). The replacement of zebra mussels by quagga mussels in the Great Lakes in the 1990s–early 2000s led to an order-of-magnitude increase in lake-wide dreissenid population density and biomass (22 g/m^2^ during zebra mussel maximum vs. 403 g/m^2^ during quagga mussel maximum, averages over lakes Huron, Ontario, and Michigan; Table 2, Fig. 1) and a shift in the bulk of mussels from nearshore to deeper areas (Fig. 2).

In addition, the spatial distribution differs between *Dreissena* species and largely depends on lake morphometry (Fig. 1, 2). In polymictic lakes, both species can potentially colonize the entire bottom. In contrast, in stratified lakes zebra mussels are largely limited to the shallow warm littoral zone while quagga mussels can colonize the whole bottom down to the deepest part (Fig. 2). There was also a substantial difference in quagga mussels spatial distribution between the inland stratified lakes and the Great Lakes (Fig. 2). In the latter, the bulk of mussel biomass was in the 20–50 m depth zone and the maximum shifts deeper over the course of invasion, while in the inland stratified lakes, the quagga mussel maximum was in the shallow (< 20 m) depth and sharply declined with depth (Fig. 2). Overall, in inland stratified lakes over 60% of quagga mussel biomass was in the warm nearshore zone, while in the Great Lakes about 83–93% of mussels were found in the cold profundal zone 20 years after invasion detection.

In all studied inland lakes, the overall lake-wide quagga mussel biomass always exceeds that of zebra mussels (average for all lakes where both species were present, 666 ± 132 vs. 279 ± 120 g/m^2^), and the difference was larger in stratified than in polymictic lakes. Regardless of lake type, in all waterbodies analyzed in this study, quagga mussels form higher biomass than zebra mussels and outcompete them (Table 2). Therefore, later in the invasion, *Dreissena* ecosystem impacts manifest almost exclusively through the quagga mussel in all 14 lakes invaded by both species.

### Lake size affects the timing and magnitude of the Dreissena impact

**Fig. 3:**
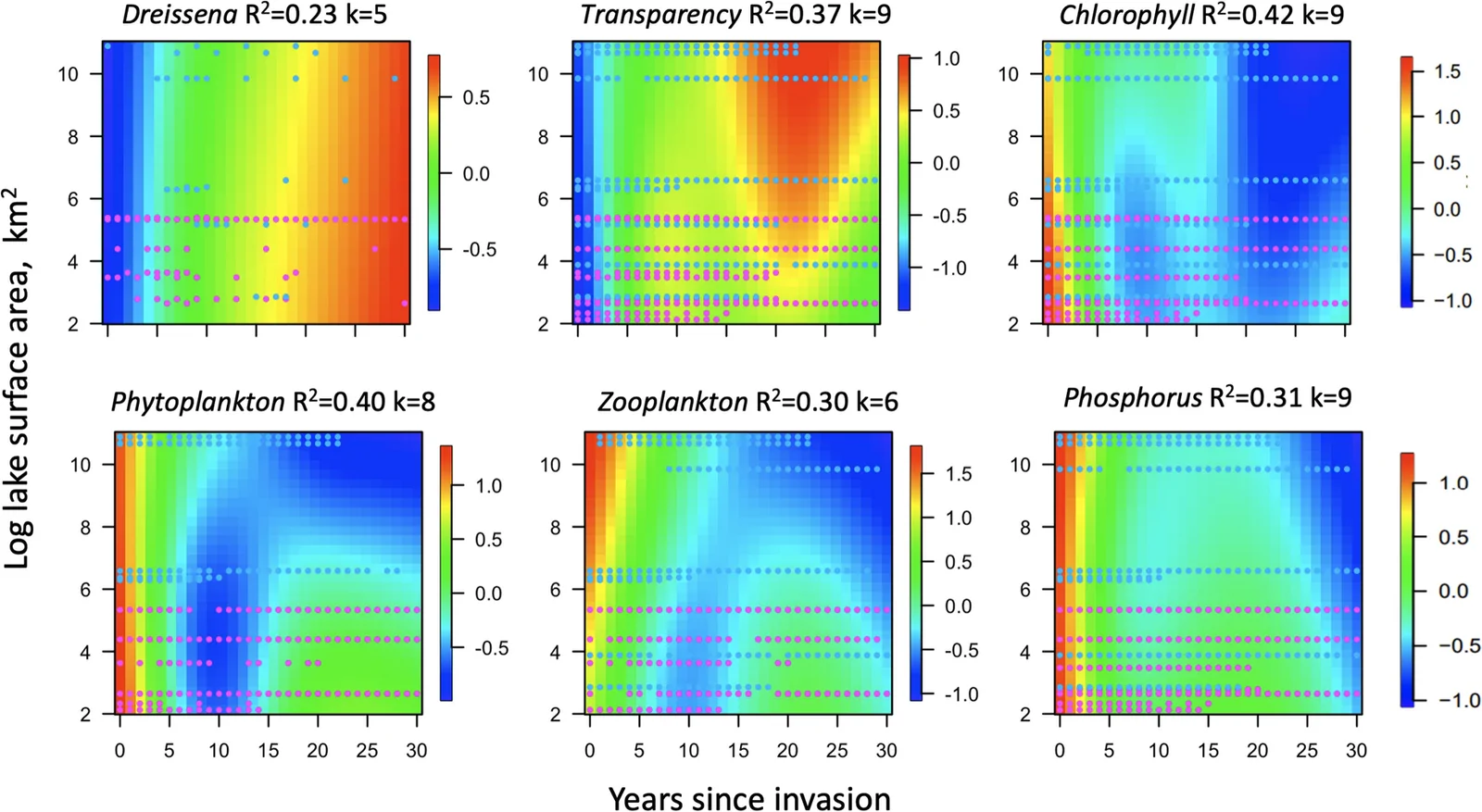
Heatmaps showing trends in each ecosystem variable (z-scores, colors) over years since invasion (x-axes) and log lake area (y-axes), derived from a 2-d spline fitted to data from 18 lakes. Dots denote observations for each lake (each lake has a distinct area); pink dots denote polymictic, blue—stratified waterbodies. k-values denote the flexibility (basis dimension) of the best-fit splines

**Fig. 4:**
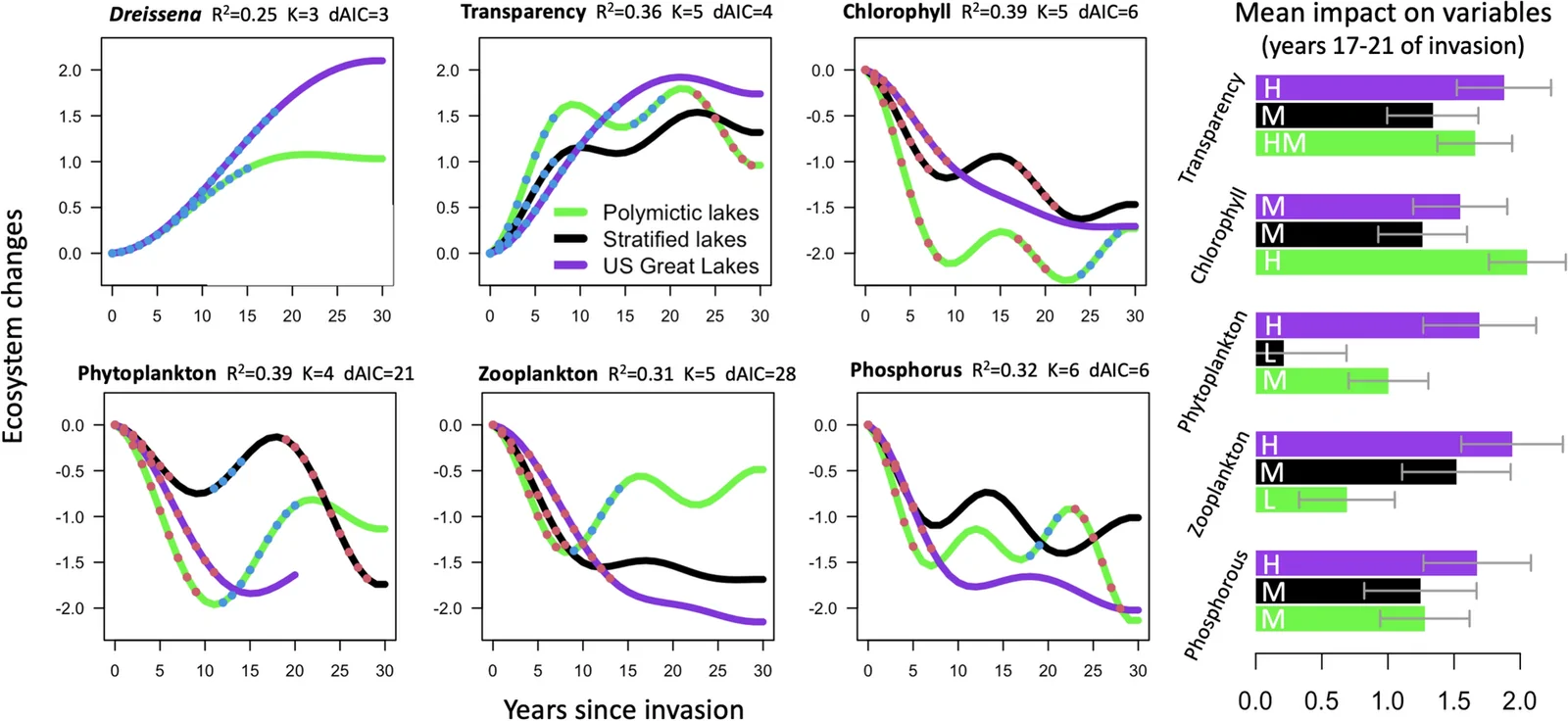
Long-term changes in ecosystem variables across 18 lakes in Europe and North America after *Dreissena* invasion. Time plots: lines show mean z-score changes in variables predicted by splines across years since invasion (x-axes), fitted hierarchically to three types of lakes: polymictic (green), stratified (black), and the Great Lakes (purple). For each variable, dot colors denote significant increases in blue and significant decreases in red. For each variable, “dAIC” denotes the improvement of models accounting for lake size over models without lake size as a factor, with larger values denoting larger differences among lake sizes. *R*^*2*^ values denote the proportion of total variance explained by fitted models; *k* values denote the basis dimension (maximum smoothness) of fitted splines (see Methods). Bar plot: mean ± 97.5% confidence intervals of mussel impacts on each ecosystem variable. Impact (H—high, L—low, and M—medium impact) is the absolute value of ecosystem change in the time plots, averaged over years 17–21 since invasion. Bars with different letters indicate significant differences (*p *< 0.025) in impact among lake size groups. The purple line is trimmed for phytoplankton, and the black line is absent for *Dreissena* due to a lack of data

**Fig. 5:**
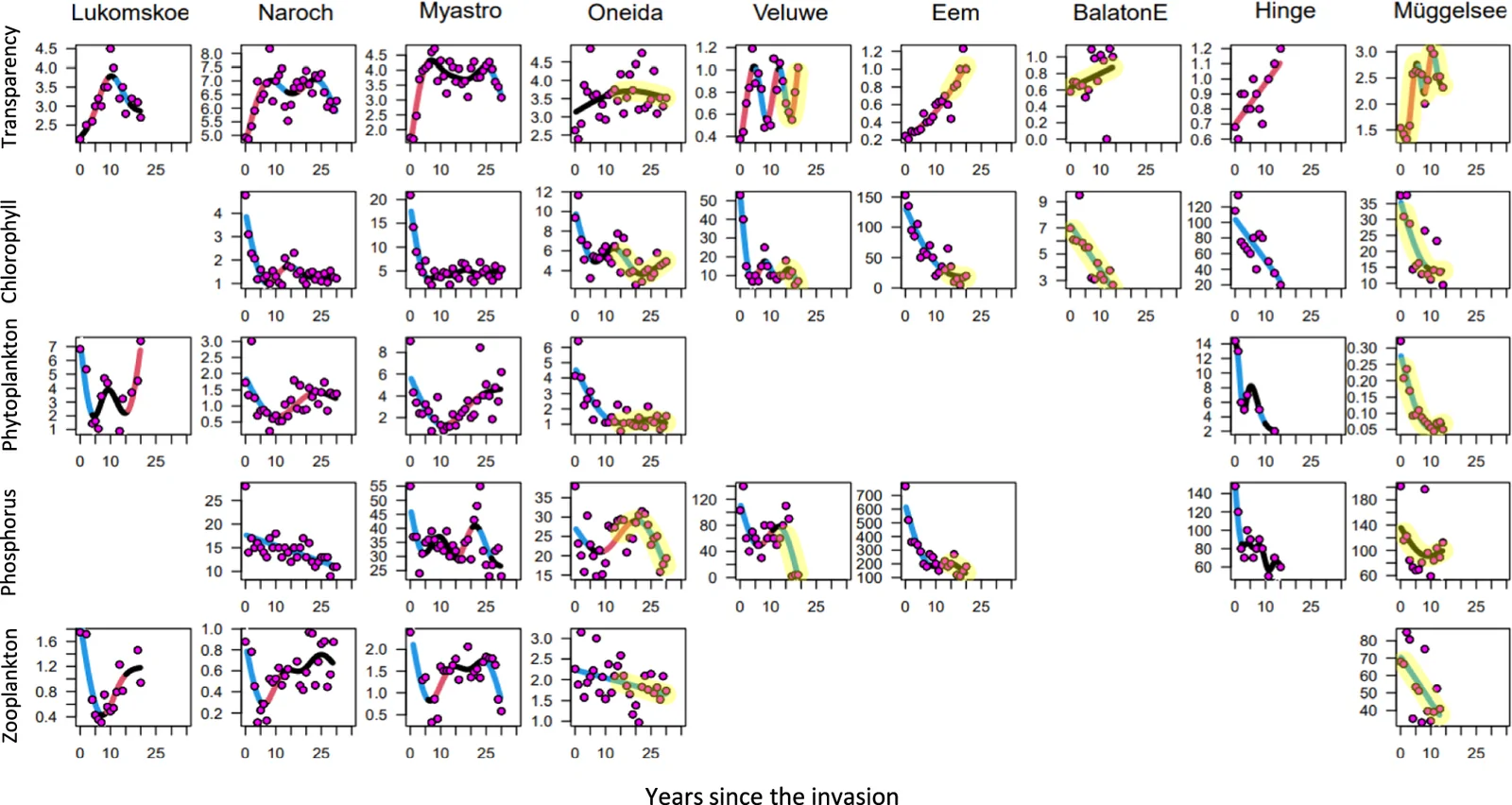
Long-term dynamics of transparency (Secchi depth, m), chlorophyll *a* concentration (μg/L), wet phytoplankton biomass (g/m^3^), total phosphorus (μg/L), and wet zooplankton biomass in polymictic lakes. Zooplankton was measured as density in Lake Müggelsee (including rotifers) and as wet biomass (g/m^3^) in all other lakes. Colors denote significance: blue indicating a significant decline and red a significant increase. Lines highlighted in yellow represent the effects unfolding after the invasion of quagga mussels

**Fig. 6:**
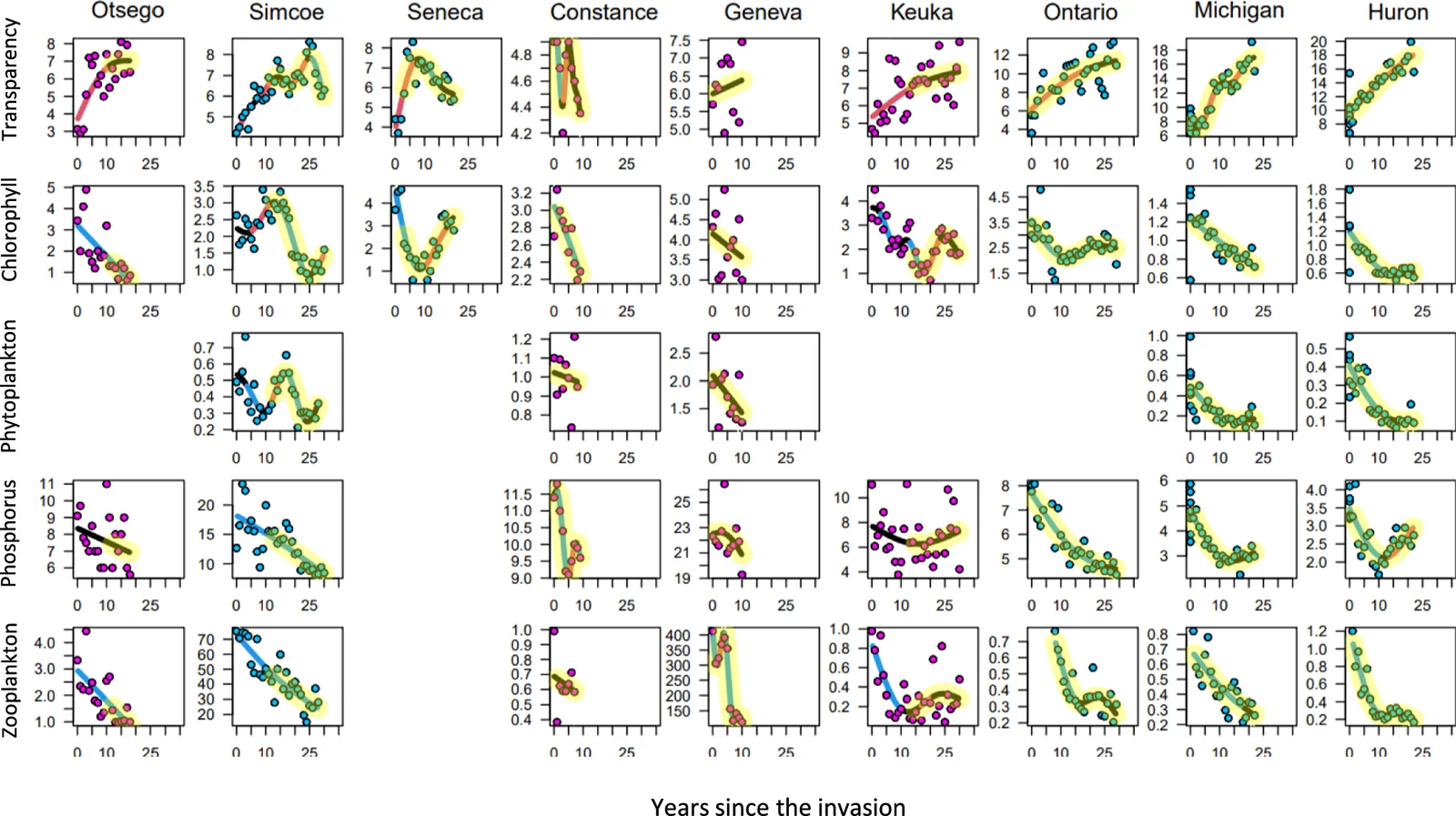
Long-term dynamics of transparency (Secchi depth, m), chlorophyll *a* concentration (μg/L), wet phytoplankton biomass (g/m^3^), total phosphorus concentration (μg/L), and zooplankton in inland stratified lakes and Great Lakes. Zooplankton was measured as density in lakes Simcoe (crustaceans only), Constance, and Geneva (crustaceans and rotifers in both lakes), and as wet biomass (g/m^3^) in all other lakes. Colors denote a significant decline in blue and a significant increase in red. Lines highlighted in yellow represent the effects of quagga mussels

During the initial stage of the invasion, we found significant changes in every measured ecosystem variable (Figs. 3, 4, 5, 6) across almost all of the18 lakes spanning a broad range of lake size, depth, retention time, and mussel biomass. These changes included significant increases in Secchi depth (83%, 15 of 18 lakes) and significant declines in chlorophyll (88%, 15 of 17), phytoplankton (82%, 9 of 11), zooplankton (92%, 12 of 13 lakes), and total phosphorus (73%, 11 of 15 lakes; Figs. 5, 6). However, the magnitude and timing of impacts varied significantly with lake size: that is, models with lake size class-dependent impacts outperformed models omitting lake size. Significant effects of lake size were found for all ecosystem variables (ΔAIC > 2, Fig. 4), and especially phytoplankton and zooplankton (ΔAIC > 20).

**Fig. 7:**
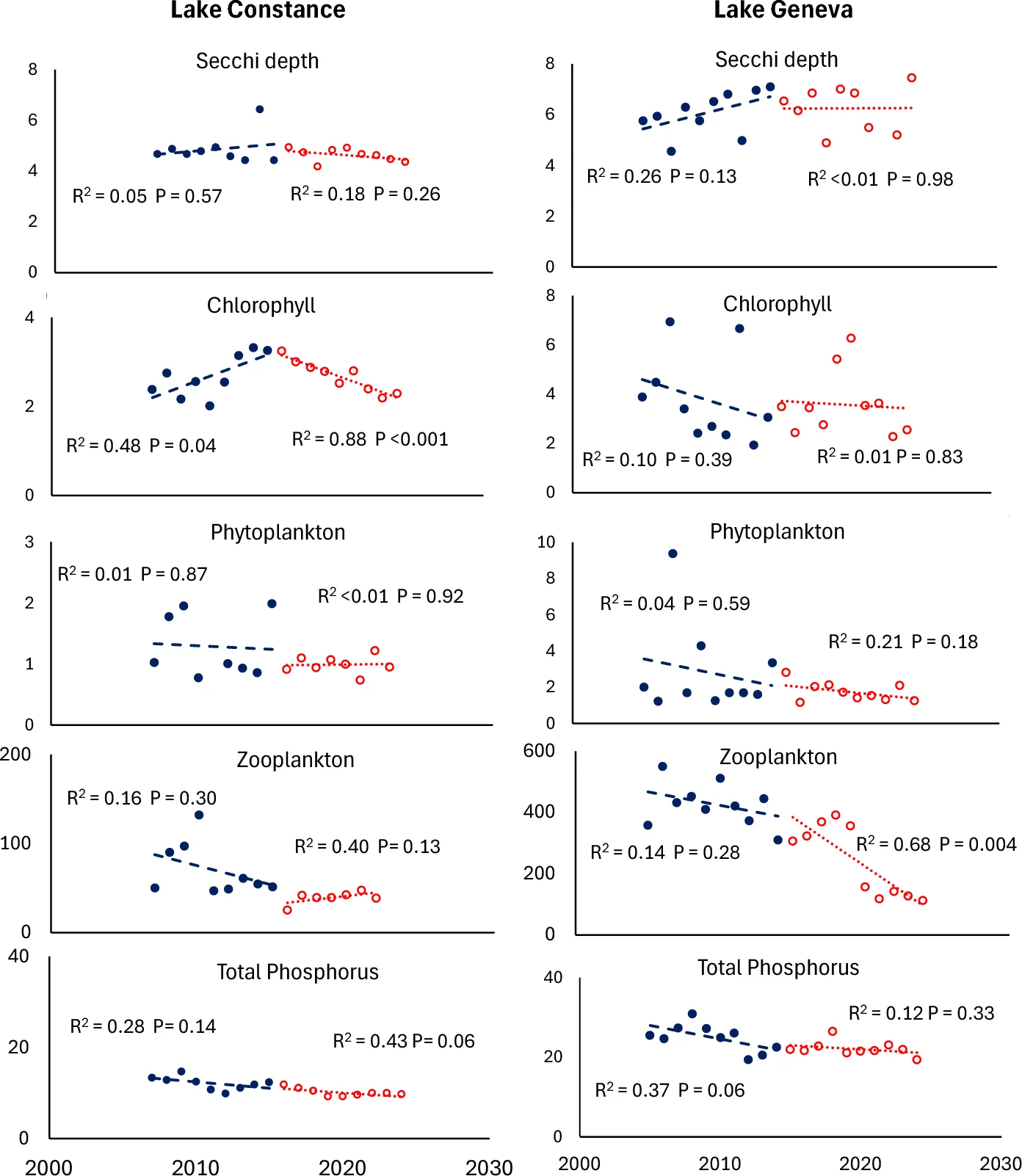
Long-term dynamics of transparency (Secchi depth, m), chlorophyll *a* concentration (μg/L), wet phytoplankton biomass (g/m^3^), zooplankton density, and total phosphorus concentration (μg/L) in lakes Constance and Geneva (Online Resource 1). Blue-filled circles and blue dashed lines denote pre-quagga mussel invasion detection, and open red circles and red dotted lines denote post-invasion detection

The timing of the response differed significantly among all lake types (Fig. 3). In polymictic lakes initially colonized by zebra mussels, the ecosystem impact manifested within 3–10 years of the initial invasion, depending on the lake and environmental variables (Fig. 5). Lake Müggelsee demonstrated a similar ecosystem response to invasion by quagga mussels to other polymictic lakes despite being originally invaded by zebra mussels in the nineteenth century. Stratified inland lakes showed generally similar ecosystem responses to *Dreissena* invasion, although lakes Constance and Geneva exhibited less consistent and sometimes opposing responses to quagga mussel invasion compared to the other lakes (Figs. 6, 7). In contrast, in the Great Lakes, quagga mussels reached maximum population biomass 3–4 times slower than in inland lakes (Table 2), resulting in a 12–20 year delay in their ecosystem impacts (depending on the environmental parameter; Figs. 3, 4, 6).

The magnitude of *Dreissena* invasion impacts was highest in the Great Lakes across all variables except chlorophyll, which declined most in polymictic lakes. As we describe next, this difference arises largely from the absence of ecosystem recovery in the Great Lakes. Together, the longer and greater impacts on the Great Lakes compared with those on smaller lakes indicate that lake size was more important than stratification in determining the impacts of *Dreissena*.

### Ecosystem recovery slows or disappears in larger lakes after quagga mussel invasion

In polymictic lakes invaded only by zebra mussels, plankton communities partially recovered (defined as a partial return to pre-invasion conditions) 8–15 years after the initial invasion (Figs. 3, 4, 5). In lakes Lukomskoe, Naroch, and Myastro, a clear and significant recovery was observed for most parameters analyzed (Figs. 3, 5); no clear recovery was observed in Lake Hinge, possibly due to the short, less consistent observations. In addition, some recovery was observed in Oneida Lake and Lake Veluwe during the zebra mussel invasion. The recovery following zebra mussel invasion was interrupted, and ecosystem impacts were amplified in lakes where quagga mussel invasion followed zebra mussel invasion, further depleting chlorophyll concentrations, phytoplankton and zooplankton abundance, and increasing Secchi depth (Figs. 3, 4, 5). We did not detect ecosystem recovery from this second invasion by quagga mussels in polymictic lakes, likely due to the short duration of observations.

Limited signs of ecosystem recovery were also observed in three of six stratified inland lakes analyzed (Fig. 6). Lake Simcoe, having the longest and most detailed data among stratified lakes, showed a significant decline in Secchi depth and increases in chlorophyll and phytoplankton after 25 years of *Dreissena* invasion (Fig. 6). In Keuka Lake, zebra mussel impacts peaked about 7 years after the initial invasion, especially evident in chlorophyll. These impacts were then amplified by the invasion of the quagga mussel, followed by partial recovery evident in chlorophyll, zooplankton, and total phosphorus (although nonsignificant for the last variable). This recovery was recorded about 20 years after the zebra mussel invasion and only 5 years after the quagga mussel invasion. In addition, in Seneca Lake, after 10 years of invasion, Secchi depth declined and chlorophyll increased.

No signs of recovery have been observed in the Great Lakes (Figs. 3 and 6). The only exception was a significant increase in total phosphorus in Lake Huron about 20 years after the first record of quagga mussels in the lake. Lake Huron has the lowest *Dreissena* biomass among the four lower Great Lakes. Note that not all variables were studied in all lakes, and not all lakes had sufficiently long data to detect recovery. Taken together, the long-term effects of mussel invasion were weakest in polymictic lakes and strongest in the Great Lakes.

## Discussion

Dreissenids strongly affect all ecosystem variables tested in this study, and these effects manifest quickly after colonization (< 10 years after invasion), except in the Great Lakes (10–20 years after invasion). Impacts after the initial *Dreissena* invasion were consistently observed across lake types and *Dreissena* species, but the timing and magnitude of these impacts were governed by lake size (Fig. 4). Later in the invasion, the outcomes of *Dreissena* impacts were less consistent (Fig. 4). Partial ecosystem recovery observed after zebra mussel invasion in inland lakes was less evident for quagga mussel invasions. In the Great Lakes, the impacts of dreissenids on ecosystems were slower to manifest because mussel populations took longer to reach their maximum biomass. Furthermore, no ecosystem recovery in the Great Lakes has been observed to date, even 30 years after mussel invasions began. However, the lack of ecosystem recovery could be confounded by other factors, including the fact that concerted efforts have been made to reduce nutrient loading into the Great Lakes. Consequently, the Great Lakes experienced greater overall changes in most ecosystem variables measured, particularly for phytoplankton and zooplankton.

The peak of ecosystem impacts and *Dreissena* biomass were often not synchronized. In polymictic lakes, impacts manifested substantially later than the peak of mussel biomass; in inland stratified lakes, they were nearly synchronized, whereas in the Great Lakes, impacts preceded the peak of mussel biomass. Our results indicated that lake size (area) is more important than other parameters in determining the impact of *Dreissena*, and that findings from smaller inland lakes cannot be directly applied to larger systems (e.g., the Great Lakes) without appropriate adjustment, and vice versa.

### Lake morphometry governs Dreissena population growth and distribution

The magnitude and extent of *Dreissena* ecosystem impacts depend on several factors, including mussel abundance, spatial distribution, water mixing rates, and access to the pelagic environment (Karatayev et al. 2002; Reed-Andersen et al. 2000; Kelly et al. 2010; Karatayev and Burlakova 2025a). These factors, however, vary dramatically among lake types, time since the initial invasion, and *Dreissena* species (Figs. 1, 2). Early in the invasion, both *Dreissena* species usually colonize the shallow zone of lakes and then spread deeper, depending on the lake type. In well-mixed polymictic lakes, both *Dreissena* species are generally limited to the shallow zone with abundant hard substrate for mussel attachment (Burlakova et al. 2012; Goedkoop et al. 2011; Karatayev and Burlakova 2025a). In the profundal zone of polymictic lakes with soft sediments, dreissenids are limited by the lack of appropriate substrates for attachment and by wave action, which inhibits colonization of unconsolidated substrates. Therefore, the spatial distribution of *Dreissena* in polymictic lakes is usually skewed to the shallow littoral zone with abundant hard substrates, although deviations from this trend arise as quagga mussels form higher densities on soft sediments in sheltered areas and zebra mussels dominate in most shallow areas exposed to strong wave activity (Fig. 2; reviewed in Karatayev and Burlakova 2025a). Nevertheless, in polymictic lakes, both dreissenid species have a strong and continuous impact on pelagic communities through consistent access to the entire water column.

In stratified inland lakes, early in the invasion, the bulk of biomass for both species is also skewed toward the shallowest zone; however, over time, quagga mussels move deeper and colonize the entire bottom, provided that oxygen limitation in the deepest zones does not prevent this (Fig. 2). Yet their density in the deep profundal zone is usually lower than in the littoral zone. Overall, in stratified lakes, quagga mussels attain much higher population densities than zebra mussels and outcompete them to near extinction, thereby largely determining their ecological impacts.

A dramatic difference in the distribution patterns of both dreissenid species was observed between the deep Great Lakes and all inland lakes. In the profundal zone of lakes Ontario, Michigan, and Huron, quagga mussels colonize the entire bottom down to the maximum depths (> 200 m), with current peak densities at 20–100 m. They attain a population biomass exceeding that of zebra mussels by an order of magnitude and virtually extirpate the latter (Fig. 2; Nalepa et al. 2010; Karatayev et al. 2021; Karatayev and Burlakova 2025b). In shallow areas (< 20–30 m), dreissenids in the Great Lakes are inhibited by strong wave activity, especially in sandy areas, limiting mussels to hard substrates (boulders, rocks, bedrock) (Fig. 2; reviewed in Karatayev and Burlakova 2025b). The 30 m threshold roughly separates the bottom of these lakes into well-mixed, warm areas with high mussel growth (< 30 m) and a deeper, more stable cold zone. Early in the invasion, the bulk of mussel biomass (65–99%) was located above 30 m, but over time, the bulk of biomass (> 80%) shifted below the thermocline, with a maximum at 30–100 m. This mid-depth maximum of quagga mussel occurred at depths where the pre-mussel benthic nepheloid layer (BNL)—a layer of particulates above the bottom characterized by elevated levels of particle density and total phosphorus—was used to form (Barbiero et al. 2024). The high concentration of suspended particles in the BNL that support both high biological activity and include living and detrital biological material (Baker and Eisenreich 1989), was likely the reason why quagga mussels were able to reach high densities at 30–100 depth intervals. However, the high filtration activity of quagga mussels eventually led to the disappearance of BNL, resulting in a decline in mussel growth later in the invasion (Karatayev et al. 2018a, 2021; Barbiero et al. 2024; Karatayev and Burlakova 2025b).

Thus far, this pattern has not been observed in the inland stratified lakes. Although we expect mussel biomass to increase over time in the deeper areas of lakes Constance and Geneva, it is likely that a large portion of biomass in these lakes will remain in the shallowest areas, as wave activity is less severe in these lakes compared to the Great Lakes, allowing mussels to form high densities. Another substantial difference between the Great Lakes and inland lakes is mussel population growth. On average, it takes 3–4 times longer for quagga mussels to reach maximum biomass in the Great Lakes than in polymictic and inland stratified lakes, thereby delaying their ecological impact.

### Lake size affects the timing and magnitude of the Dreissena* impact*

Early in the invasion, when *Dreissena* populations grew exponentially, their impacts on invaded waterbodies were high, and most impacts were direct (Karatayev et al. 1997, 2023; Strayer et al. 2019). At this stage of invasion, the general pattern of ecosystem response was consistent across lake types, despite large differences in lake size, depth, retention time, and *Dreissena* biomass. Common ecosystem responses across invaded lakes included increases in Secchi depth, declines in chlorophyll, phytoplankton, and zooplankton biomass, and total phosphorus (Fig. 3). These results were expected, as they are well described in the literature (reviewed in Karatayev et al. 2002; Higgins and Vander Zanden 2010; bij de Vaate et al. 2014; Karatayev and Burlakova 2025b). However, in addition to these well-known patterns, we show that both the timing of the initial response and the degree of ecosystem recovery differed significantly among lake types (Figs. 3, 4, 5, 6).

We found that lake morphometry, especially lake size, governs the timing and magnitude of *Dreissena* ecosystem impacts. Although the general pattern of ecosystem response to *Dreissena* invasion was similar across inland polymictic and stratified lakes, the timing of the effect was different. In polymictic lakes initially colonized by zebra mussels, ecosystem impacts manifest within 3–10 years of invasion (Figs. 3, 5). In stratified lakes, zebra mussel impacts manifest with a small but significant difference within the first 4–12 years of invasion (Fig. 4). The timing of *Dreissena* ecosystem impacts is not always synchronized with their population maxima; they are delayed in polymictic lakes but occur nearly simultaneously with population peaks in stratified inland lakes (Table 2, Figs. 3, 4, 5, 6). In our dataset, in almost all polymictic lakes, the initial ecosystem response was driven by zebra mussel impacts, which peaked 2–3 years after the initial invasion, including the first year, when the population was very sparse. We hypothesize that this period was not long enough to reach maximum impact, resulting in a delayed ecosystem response. In the inland stratified lakes, *Dreissena* ecosystem impacts largely manifested through quagga mussel. Their population reached a maximum 6–7 years after the initial invasion—long enough to cause maximum ecological impacts.

In the Great Lakes, *Dreissena* ecosystem impacts manifest significantly later than in inland lakes, 12 to 20 years after quagga mussel invasion (Figs. 3, 4, 5, 6), and precede the lake-wide peak in mussel biomass, which occurs 20–28 years after initial invasion. Early in the invasion of the Great Lakes, the majority of *Dreissena* occurred in the well-mixed nearshore zone, where an abundant food supply coupled with warm temperatures during the growing season results in high mussel growth, short longevity, and strong ecological impact per unit of mussel biomass (Karatayev et al. 2018a, 2021; Elgin et al. 2022; Karatayev and Burlakova 2025b). Later in the invasion, the bulk of dreissenids moved to the deep profundal zone where depleted food availability and lower temperatures resulted in slow growth and long lifespans (Karatayev et al. 2018a; Elgin et al. 2022). For example, the annual growth of initially 12-mm quagga mussels in cage experiments in Lake Ontario declined from 10.2 ± 0.8 mm at 15 m depth to 5.9 ± 0.2 mm at 45 m, and to 0.7 ± 0.4 mm at 90 m depth (Elgin et al. 2022). These deep-dwelling dreissenids become less physiologically active, access the epilimnion only during periods of isothermal conditions, and therefore have a weaker impact on pelagic communities. Therefore, the shift of the bulk of mussel biomass deeper into the profundal zone leads to a buildup of mussel biomass that has limited impact on the epilimnion during the stratified period, and the maximum impact of the quagga mussel ecosystem in the Great Lakes often precedes the population maximum.

### Ecosystem recovery slows or disappears in larger lakes after quagga mussel invasion

Partial ecosystem recovery of planktonic communities following zebra mussel invasion, observed in polymictic lakes in our previous study (Karatayev et al. 2023), was also evident in stratified lakes (e.g., Lake Simcoe, Keuka, and Seneca). In Lake Simcoe, phytoplankton recovered about 10 years after zebra mussel invasion, declined following quagga mussel introduction, and then partially recovered again approximately 12 years after the quagga mussel invasion. This recovery has been observed for Secchi depth and chlorophyll but not yet for zooplankton or total phosphorus (Fig. 5). In Keuka Lake, a significant partial recovery of chlorophyll and zooplankton began 20 years after the zebra mussel invasion and about 5 years after the quagga mussel invasion. In Seneca Lake, the initial introduction of zebra mussels in 1992 and quagga mussels in 1994 was associated with a 2-fold increase in Secchi depth and an almost 6-fold decline in chlorophyll concentration, but the trend reversed after a major die-off of mussels in 1998, resulting in a decline in Secchi depth and an increase in chlorophyll concentrations (Hoering and Halfman 2010; Halfman et al. 2010).

Despite more than 30 years of observation, no clear signs of ecosystem recovery have yet been detected in the Great Lakes. However, recent stabilization of dreissenid biomass in Lakes Ontario, Michigan, and Huron (Fig. 1; Karatayev and Burlakova 2025c) may allow ecosystem recovery in the coming decades. This suggestion is supported by the observation that the decline in planktonic community abundance and phosphorus levels is slowing and, in most cases, has leveled off. In addition, in Lake Huron, which harbors the smallest *Dreissena* population, phosphorus concentrations increased significantly 20 years after invasion (Fig. 6).

### Quagga mussels have stronger impacts than zebra mussels, especially in stratified lakes

Historically, most information on *Dreissena* ecological impacts was based on the zebra mussel (reviewed in Karatayev et al. 2015; Karatayev and Burlakova 2025a) or on dreissenids in general, as these two species are often considered to have very similar ecological impacts and are therefore treated jointly (Higgins and Vander Zanden 2010; Kelly et al. 2010; Kissman et al. 2010). However, the lag time for zebra mussels between their first record in the lake and when the population reaches a maximum is shorter than that for quagga mussels (Table 2; Karatayev et al. 2011, 2015; Hetherington et al. 2019), suggesting that zebra mussel ecosystem impacts will manifest earlier. On the other hand, quagga mussels utilize a wider array of habitat types because they colonize rocky shallows as well as soft-sediment habitats in deep areas of lakes, allowing them to attain higher lake-wide biomass than zebra mussels. In stratified lakes in particular, where zebra mussels are largely limited to the well-mixed nearshore environment, quagga mussels can colonize the entire bottom, reaching biomass levels exceeding zebra mussels by an order of magnitude (Table 2; Fig. 2; Karatayev et al. 2015, 2021; Karatayev and Burlakova 2025b). In addition to higher total mussel biomass, quagga mussels also utilize resources more efficiently across seasons by actively filter-feeding during cold periods (Haltiner et al. 2023; Karatayev et al. 2023). Therefore, the timing and magnitude of *Dreissena* ecosystem impacts should depend on the time since the initial invasion, the *Dreissena* species, and lake morphometry. Indeed, the invasion of quagga mussels into lakes already colonized by zebra mussels amplified the initial impacts of *Dreissena*, as seen in both polymictic and stratified lakes (Figs. 5, 6). In polymictic Oneida Lake, compared with years of zebra mussel dominance (1993–2007), quagga mussel dominance in 2009–2017 corresponded to significantly greater water clarity and lower chlorophyll concentrations during early spring and late fall, allowing them to have a stronger impact on the invaded ecosystems per unit of mussel biomass (Karatayev et al. 2023). Similarly, in stratified Lake Simcoe, quagga mussel invasion was associated with significantly greater impacts on all environmental variables (Fig. 6). The ability to identify the effects of serial invasion in both cases likely reflects the fact that Oneida Lake and Lake Simcoe are the best-studied lakes within their lake types and have the longest monitoring records.

The most pronounced difference in ecosystem response between zebra and quagga mussel invasions occurs in the Great Lakes, where zebra mussel effects were primarily nearshore (Hecky et al. 2004) and more limited at the lake-wide scale, while quagga mussels fundamentally altered ecosystem structure and function (i.e., Vanderploeg et al. 2002). The stronger ecosystem impacts of quagga mussels, which manifest later than those of zebra mussels, may help explain the limited evidence of ecosystem recovery in lakes invaded by quagga mussels, particularly in the Great Lakes, compared with inland lakes colonized by zebra mussels. Continued long-term monitoring is needed to determine if and when ecosystems affected by quagga mussels show signs of recovery.

### Results robustness

Eighteen lakes analyzed in this study span a >7000-fold range in lake area, an almost 130-fold range in mean lake depth, from small polymictic lakes to one of the largest lake systems in the world, the North American Great Lakes (Tables 1, 2). Other differences include geographical regions, *Dreissena* biomass, and the dates of the initial invasion. Nevertheless, the general pattern of ecosystem response during the initial invasion (the first 10 years in inland lakes and the first 20 years in the Great Lakes) was remarkably similar across all lakes (in 15 to 17 of the 18 lakes, depending on the variable) (Figs. 3, 4, 5, 6). However, these data can be used to predict dreissenid invasion impacts only with caution, as any single system may deviate from aggregate lake trends due to system-specific trajectories in mussel biomass, abiotic factors mediating mussel impacts, and other factors affecting the lake ecosystem (e.g., changes in land use and agricultural practices; climate change; re-oligotrophication in some cases). This emphasizes the necessity of evaluating the ecological impacts of invaders at the population rather than species level (Sousa et al. 2024).

Additionally, the two Alpine lakes Constance and Geneva deviate substantially from the overall trends we detect, showing no increases in Secchi depth or decreases in phytoplankton – two of the most common outcomes of *Dreissena* invasion (Higgins and Vander Zanden 2010; Goedkoop et al. 2011; Mayer et al. 2014; Karatayev and Burlakova 2025a, b; Fig. 7). In addition, no significant declines were found in chlorophyll and total phosphorus in Lake Geneva and in zooplankton in Lake Constance. This is despite the fact that the current average quagga mussel population biomass in these lakes is similar (Lake Constance) or higher (Lake Geneva) than in other stratified lakes (Table 2) and the fact that mussels have been present for at least 9–10 years, long enough to observe the strongest ecosystem changes in other stratified lakes in our dataset. Both lakes have retention times comparable to those of other lakes (Table 1), suggesting that other factors may be responsible for the unusually low ecosystem impacts.

Both lakes experienced severe cultural eutrophication that peaked in the 1970s, followed by effective phosphorus-control programs (Vonlanthen et al. 2012). These efforts resulted in a dramatic reduction in phosphorus and declines in phytoplankton and zooplankton (Fig. 7). These processes of re-oligotrophication, resembling dreissenid benthification effects (Mayer et al. 2014), make it difficult to separate the roles of these two factors. Additionally, in recent decades, both lakes have become largely meromictic (lakes in which water layers do not mix, leading to permanent stratification), reducing the deep-water quagga mussel population's access to the epilimnion. Thus, due to global warming, Lake Geneva's mixing remained incomplete for twelve consecutive years (CIPEL 2025), and Lake Constance mixed to the bottom only twice in the last 30 years (IGKB 2025). In this context, occasional mixing that releases phosphorus from the hypolimnion may be a major environmental factor masking the impacts of quagga mussels (and the overall trend in re-oligotrophication). For example, incomplete mixing in Lake Constance in 2014 led to low bottom oxygen concentrations and enhanced phosphorus release from sediments, resulting in pronounced peaks in phosphorus and chlorophyll in 2014–2015 that largely shaped ecosystem trends over the subsequent decade (Fig. 7). The significant decline in chlorophyll concentration in Lake Constance after 2015 may, in part, be attributed to the impact of quagga mussels. Additionally, the somewhat stronger decline in zooplankton observed over the past decade in Lake Geneva, compared with the preceding nine years, may indicate effects of the quagga mussel invasion. However, so far, the impacts of quagga mussels on the epilimnion in lakes Constance and Geneva are less evident than in other waterbodies (Figs. 4, 5, 6, 7), perhaps because the impact of quagga mussels in lakes Constance and Geneva occurred simultaneously with other ecosystem drivers associated with re-oligotrophication and changes in mixing regimes related to climate change. The effects of inconsistent mixing may be more important than effects of mussels on these ecosystems, but their importance will depend on interactions among increased temperature, changes in wind patterns, and individual lake bathymetry. Lake-specific hydrodynamic models (e.g., Richardson et al. 2017; Bruce et al. 2018) combined with mussel distributions will likely be necessary to predict mussel effects in these changing ecosystems.

The decline in phytoplankton typical of early dreissenid invasion was also not significant in Lake Ontario, suggesting that the negative impacts of mussel filter feeding on phytoplankton were offset by other factors, such as increased nutrient loading or *Dreissena*'s limited access to the epilimnion. A discrepancy in the responses of chlorophyll and turbidity after *Dreissena* invasion was observed in the western basin of Lake Erie and was attributed to high sediment loads entering the basin and the resuspension of unassimilated non-algal particulates (Barbiero and Tuchman 2004). In addition, *Dreissena* impacts can be reduced by pollution or hypoxia that kill or even extirpate mussels, as observed for quagga mussels in the central basin of Lake Erie (Barbiero and Tuchman 2004; Karatayev et al. 2018b), quagga mussels in Hamilton Harbor of Lake Ontario (Burlakova et al. 2025), zebra mussels in Lake Lukomskoe in 2005 (Mitrakhovich et al. 2008), and zebra mussels in Dutch lakes in the 1970s (Ibelings et al. 2007; Noordhuis et al. 2016), or dreissenid effects might be masked by external factors causing the response variable to increase.

## Conclusions

During the initial stage of the invasion, *Dreissena* caused significant changes in every ecosystem variable measured across a broad range of lake sizes, depths, retention times, and mussel biomass, including increases in Secchi depth and declines in chlorophyll, phytoplankton, and zooplankton biomass, as well as in total phosphorus. The timing of the response differed significantly among all lake types, with the largest difference between inland lakes and the Great Lakes.

In polymictic lakes initially colonized by zebra mussels, ecosystem impacts manifest 3–10 years after the initial invasion, depending on the lake and environmental variables. In stratified lakes, zebra mussel impacts emerge within the first 4–12 years of invasion, with a small but significant difference compared to polymictic lakes. In the Great Lakes, *Dreissena* reached maximum population biomass three to four times later than in inland lakes, explaining a large delay in ecosystem impacts that manifest 12–20 years after introduction. Dreissenid ecosystem impacts are not always synchronized with *Dreissena* population maxima: impacts are delayed in polymictic lakes, closely coincide with population peaks in stratified inland lakes, and manifest earlier in the Great Lakes.

Following initial invasion impacts, plankton communities in polymictic lakes did not stabilize at a lower level; instead, they partially recovered 8–15 years after the initial invasion in three of four zebra mussel-invaded ecosystems. In stratified lakes, signs of ecosystem recovery were observed, but only in lakes with the longest and most detailed monitoring data. No ecosystem recovery has been observed in the Great Lakes to date, despite over 30 years of monitoring. However, stabilization of dreissenid biomass in lakes Ontario, Michigan, and Huron may signal the onset of ecosystem recovery in the coming decades.

Overall, quagga mussels have stronger ecosystem impacts than zebra mussels, but the difference ranges from relatively small in polymictic lakes to extremely large in the Great Lakes, where quagga mussels have fundamentally altered entire ecosystems and now represent the dominant environmental driver in the Great Lakes (Bunnell et al. 2014; Li et al. 2021; Karatayev and Burlakova 2025b). Our results indicated that lake size is more important than other parameters in determining *Dreissena* impact, and findings from smaller inland lakes cannot be directly applied to larger systems (e.g., the Great Lakes) without appropriate adjustment, and vice versa.

## Supplementary Information

Below is the link to the electronic supplementary material.

Supplementary file1 (XLSX 51 kb)

Supplementary file2 (TXT 12 kb)
